# Victimization, perception of insecurity, and changes in daily routines in Mexico

**DOI:** 10.1590/S1518-8787.2016050006098

**Published:** 2016-09-22

**Authors:** María Elena Ávila, Belén Martínez-Ferrer, Alejandro Vera, Alejandro Bahena, Gonzalo Musitu

**Affiliations:** I Centro de Investigación Transdisciplinar en Psicología. Universidad Autónoma del Estado de Morelos. Cuernavaca, Morelos, México; II Departamento de Educación y Psicología Social. Facultad de Ciencias Sociales. Universidad Pablo Olavide. Sevilla, Andalucía, España

**Keywords:** Crime Victims, Violence, Safety, Adaptation, Psychological

## Abstract

**OBJECTIVE:**

To analyze the relationships between victimization, perception of insecurity, and changes in routines.

**METHODS:**

The 8,170 subjects of both sexes (49.9% women and 50.1% men) aged between 12 and 60 years, selected from a proportional stratified sampling, participated in this study. The measuring instrument was an adaptation of the National Survey on Victimization and Perception of Public Security. Chi-square tests were performed.

**RESULTS:**

The results show significant differences on victimization and sex regarding perception of insecurity, restrictions on everyday activities, and protection measures. 13.1% of those interviewed claimed to have been victims of a crime in the past 12 months. 52.7% of women considered their municipality as unsafe or very unsafe. In the case of men, this percentage was 58.2%. Female victims reported significant restrictions in everyday activities when compared to non-victims. In relation to men, the percentage of victims with a high restriction of activities was higher in male victims than non-victims. In the group of victimized women, the segment of women who opted for increased measures of protection against crime was larger than expected, while those of non-victims who took less protective measures was lower than expected. These same results were observed in the group of men.

**CONCLUSIONS:**

The experience of victimization implies a greater perception of insecurity. However, the climate of insecurity is widespread in a large number of citizens. Gender differences in a high-crime environment show the importance of investigating in depth the roles of both genders in the perception of insecurity and changes in routines.

## RESUMO

**OBJETIVO:**

Analizar las relaciones existentes entre victimización, percepción de inseguridad y cambios en las rutinas.

**MÉTODOS:**

Participaron en este estudio 8,170 sujetos de ambos sexos (49.9% mujeres y 50.1% hombres) de entre 12 y 60 años, seleccionados a partir de un muestro estratificado proporcional. El instrumento de medida fue una adaptación de la Encuesta Nacional de Victimización y Seguridad Pública. Se realizaron pruebas Chi-cuadrado.

**RESULTADOS:**

Los resultados evidencian diferencias significativas en cuanto a victimización y sexo con respecto a percepción de inseguridad, restricciones de actividades cotidianas y medidas de protección. Un 13.1% de las personas entrevistadas afirmaron haber sido víctimas de un delito en los últimos doce meses. El 52.7% de las mujeres consideraron su municipio como inseguro o muy inseguro. En el caso de los hombres, este porcentaje fue de 58.2%. Las mujeres víctimas señalaron restricciones en la vida cotidiana significativas en comparación con las no víctimas. Con relación a los hombres, el porcentaje de víctimas con un alta restricción de actividades fue mayor en los hombres víctimas que en los hombres no víctimas. En el grupo de mujeres victimizadas, el segmento de mujeres que optaron por mayores medidas de protección frente a la delincuencia fue mayor de lo esperado, mientras que las mujeres no víctimas que asumieron menores medidas de protección fue menor de lo esperado. Estos mismos resultados se observaron en el grupo de los hombres.

**CONCLUSIONES:**

La experiencia de victimización lleva implícita una mayor percepción de inseguridad. Sin embargo, el clima de inseguridad se encuentra diseminado en gran cantidad de la ciudadanos. Las diferencias de género en un clima de alta criminalidad muestran la importancia de investigar a profundidad los roles de ambos sexos en la percepción de inseguridad y los cambios en las rutinas.

## INTRODUCTION

The crime rate in Mexico has increased significantly in recent years^23^. This trend has also been observed in the state of Morelos, mainly in the capital (Cuernavaca) and, in particular, in those crimes that, because of its violent nature, cause great social alarm, such as kidnapping, intentional homicide, and robbery with extortion^a^.

To assess the impact of rising crime on citizens in Mexico, a series of papers explored the perception of insecurity in different cities throughout the country, from the perspective of the victims, and the relationship of citizens with law enforcement institutions. In this sense, one of the most relevant studies was the National Survey on Victimization and Perception of Public Security (ENVIPE)^b^, in which we observe that drinking alcohol in the street, use and sale of drugs, theft and assault are the most common crimes from the perspective of the victims. These crimes are not among the most relevant in the official reports published by the Secretariat of the Interior and the Attorney General of Justice of Morelos, so we highlight the importance of such surveys where the record of crimes is exceeded.

The social impact of this climate of insecurity is reflected in the deterioration of the quality of life of citizens, and the fear of being a victim of crime, particularly in those people who have already been direct and indirect victims^10^ ^,^ ^21^. In addition, the fear is related to the inhibition of social behaviors which, in turn, increases this feeling, forming a bond difficult to break and causing changes in the lifestyle of citizens^3^. Therefore, a higher perception of insecurity of victims seems to favor not only a transformation of the social interaction patterns, but also a change in daily routines, such as avoiding leaving home or passing by dangerous locations, and establishing surveillance measures within homes^17^ ^,^ ^23^. These changes reduce the use of public places, since these areas can create uncertainty about safety. Thus, for example, approximately 36.0% of people using public transport in the metropolitan area of Mexico city say they feel unsafe or very unsafe^22^. In the survey conducted by ENVIPE^b^, the most insecure places are ATM on streets, banks, public transport and the street. However, feeling unsafe is not limited to this type of environment^5^. Braakmann^2^ and Vilalta^23^ observed a close relationship between the perception of insecurity in public transport, in the street at night and at home, so that the perception of insecurity is reflected in a general social insecurity context, in public and private spaces. Likewise, in Mexico, most disturbing crimes refer to the violation of integrity, both in public and private spaces^b^.

For this reason, the objective of this study was to analyze the relationships between victimization, perception of insecurity and changes in routines in a high crime environment.

## METHODS

In this study, 8170 subjects of both sexes participated (49.9% women and 50.1% men), living at least six years in the State of Morelos (Mexico). Regarding the age, the sample was distributed as follows: 24.0% aged 12-17, 8.0% aged 18-20; 14.0% aged 21-30; 14.0% aged 31-40; 20.0% aged 41-60; and 20.0% aged 61 or above. A proportional stratified sampling was performed on the basis of population density. Thirty-three municipalities of the state of Morelos were selected. The sample size allows predictions to be made with variables selected in this study, using a coefficient of determination of 0.05 and a power of 0.90^5^ ^,^ ^c^.

The instrument was administered individually, in interview format, by 163 interviewers trained by experts and members of the research group of the Autonomous University of the State of Morelos. They chose this application strategy of the instrument to ensure the comprehension of all items by all respondents. Interviewers were randomly assigned to the four sectors in which conventionally the 33 municipalities (north, south, east and west) were grouped. A supervisor coordinated each of the sectors created. The participants were informed of the purposes of the study and anonymity and confidentiality of the data were guaranteed. 1.20% (n = 98) of respondents refused to participate in the study. All participants who agreed to participate signed a consent form to be part of the study. In these cases, other participants using the same sampling criteria were selected. The questionnaire length was between 40 and 45 minutes. The research protocol was reviewed and approved by the Committee of Ethics of the Autonomous University of the State of Morelos.

The instrument used is adapted from the National Survey on Victimization and Perception of Public Security that was applied the years 2010, 2011 and 2012 in Mexico by the National Institute of Statistics and Geography (INEGI). The following describes the variables used in the study and their psychometric properties. To evaluate the direct victimization, the following question was asked “Have you been a victim of any crime in the past 12 months?” The question was coded with two response options (1 = Yes, 2 = No).

To evaluate the perception of safety in the municipality the following question was asked “How safe is your municipality?” The question had five response options 1 = very unsafe, 6 = very safe.

Restrictions on everyday activities were analyzed by a dichotomous scale consisting of 13 items concerning the activities that have not been done for fear of being a victim of crime. A factor analysis was performed for this study. The first factor, known as restrictions in daily life, included the following items: leave early in the morning or late in the evening; wear jewelry; walk in dark and lonely streets; visit relatives or friends that who live far away; carry cash; take a taxi; don’t carry your cell phone out of sight; carry more cash than is necessary; avoid dangerous areas of the city. The second factor, known as economic restrictions, included the following items: park your car in the street; carry credit cards; use ATM; use public transportation. The Cronbach’s alpha for this study was 0.80 for the first factor, and 0.63 for the second.

For protective measures against crime, a dichotomous scale consisting of 15 items measuring the frequency of the different protective measures against the possibility of being a victim of crime was used. The previous study obtained two factors. First, physical protective measures, referred to aspects related to: purchase and carry a weapon; install alarms in the house; hire personal security; take joint actions with the neighborhood; hire private security on the street or in the colony; buy a dog; put bars or fences and increase security on doors or windows. Second, control of personal information, included the following items: avoid giving telephone information; avoid giving passwords or personal data over the internet; do not provide information to strangers; use caller ID. The Cronbach’s alpha for this study was 0.74 for the first factor, and 0.71 for the second.

Missing values by scales or subscales were obtained using the imputation regression method. All statistical analyzes were performed using SPSS program version 20.0. To analyze the relationships between the variables under study, relationships between the variables under study, a Chi-square test was conducted from which statistically significant differences were found between groups. Also, the *phi* coefficient (φ) between both variables and Cramer’s test (V) was calculated.

## RESULTS

The results obtained for the variables victimization and perception of insecurity showed significant differences. Out of the 7,480 subjects interviewed, 978 (13.1%) claimed to have been victims of a crime in the last 12 months. Victims had an increased perception of insecurity, compared to non-victims. 55.6% of subjects who were victims of crime last year considered their municipality (town or city) as “unsafe” or “very unsafe”, while 40.2% of persons who were not victims considered their municipality as “unsafe” or “very unsafe” (Table 1).

**Table1:** Victimization and perception of insecurity in the last twelve months*.

Victim of crime		Perception of insecurity					Total

		Very unsafe	Unsafe	Regular	Safe	Very safe	
Yes	n	162	381	351	74	10	978
	% of group	16.6	39.0	35.9	7.6	1.0	100
	Corrected residuals	8.2	4.4	-4.9	-4.6	-3.5	
No	n	543	2,070	2,875	823	191	6,502
	% of group	8.4	31.8	44.2	12.7	2.9	100
	Corrected residuals	-8.2	-4.4	4.9	4.6	3.5	
Total	n	705	2,451	3,226	897	201	7,480
	% of group	9.4	32.8	43.1	12.0	2.7	100

* χ^2^ = 117.646, p < 0.001; φ = 0.125, p < 0.001; V = 0.125, p < 0.001

Results showed significant differences between males and females in the relationship between victimization and perception of insecurity. 52.7% of women considered their municipality as “unsafe” or “very unsafe”. In the case of men, this percentage was 58.2%. As shown in the corrected residuals, while the proportion of female victims who consider their municipality as “unsafe” was higher than expected, for those who were not victims, the assessment of their municipality as regular, safe and very safe was higher than expected (Table 2).

**Table 2:** Victimization by gender and perception of insecurity in the last twelve monthsa.

Victim of crime			Perception of insecurity					Total

			Very unsafe	Unsafe	Regular	Safe	Very safe	
Women^b^	Yes	n	72	186	185	39	7	489
		% of group	14.7	38.0	37.8	8,0	1.4	100
		Corrected residuals	5.6	3.0	-3.2	-3.2	-1.8	-
	No	n	232	1,001	1,461	420	92	3,206
		% of group	7.2	31.2	45.6	13.1	2.9	100
		Corrected residuals	-5.6	-3.0	3.2	3.2	1.8	-
	Total	n	304	1,187	1,646	459	99	3,695
		% of group	8.2	32.1	44.5	12.4	2.7	100
Men^c^	Yes	n	90	194	166	35	3	488
		% of group	18.4	39.8	34.0	7.2	0.6	100
		Corrected residuals	6.1	3.2	-3.7	-3.3	-3.1	-
	No	n	308	1,065	1,406	403	99	3,281
		% of group	9.4	32.5	42.9	12.3	3.0	100
		Corrected residuals	-6.1	-3.2	3.7	3.3	3.1	-
	Total	n	398	1,259	1,572	438	102	3,769
		% of group	9.4	32.8	43.1	12.0	2.7	100

^a^ χ^2^ = 117.990, p < 0.001; φ = 0.126, p < 0.001; V = 0.126, p < 0.001

^b^ χ^2^ = 52.994, p < 0.000; φ = 0.120, p < 0.001; V = 0.120, p < 0.001

^c^ χ^2^ = 66.316, p < 0.001; φ = 0.133, p < 0.001; V = 0.133, p < 0.001

Male and female victims also differed in activity restrictions for fear of being victimized. Female victims indicated significantly more restrictions in comparison with non-victims; this trend was found in the corrected residuals (Table 3). In relation to men, the percentage of victims with high activity restriction (81.3%) was higher than in male non-victims (56.9%). The least restriction of everyday activities was observed in men who were not the subject of crime in the last year (Table 3).

**Table 3:** Victimization by gender and restrictions on everyday activitiesa.

Victim of crime			Restrictions on everyday activities		Total

			Low	High	
Women^b^	Yes	n	90	131	221
		% of group	40.7	59.3	100
		Corrected residuals	-3.8	3.8	-
	No	n	825	695	1,520
		% of group	54.3	45.7	100
		Corrected residuals	3.8	-3.8	
	Total	n	915	826	1,741
		% of group	52.6	47.4	100
Men^c^	Yes	n	41	178	219
		% of group	18.7	81.3	100
		Corrected residuals	-6.9	6.9	-
	No	n	652	860	1,512
		% of group	43.1	56.9	100
		Corrected residuals	6.9	-6.9	-
	Total	n	693	1,038	1,731
		% of group	40.0	60.0	100

^a^ χ^2^ = 55.442, p < 0.001; φ = -0.126, p < 0.001; V = 0.126, p < 0.001

^b^ χ^2^ = 14.212, p < 0.001; φ = -0.090, p < 0.001; V = .090, p < 0.001

^c^ χ^2^ = 47.441, p < 0.001; φ = -0.166, p < 0.001; V = 0.166, p < 0.001

Finally, the results confirmed the relationship between sex, victimization and protective measures against crime (Table 4). The percentage of female victims who adopted protective measures against crime is higher than that of female non-victims (Table 4). Conversely, women who were not victims adopted fewer protective measures (53.5%) compared to women who were victims of a crime in the last twelve months (27.4%). This same relationship was observed in relation to men, since the percentage of men who adopted protective measures against crime was significantly higher in those who were victims (78.3%) compared to non-victims (56.4%). Therefore, as shown in the standardized corrected errors, in the victimized women’s group, the proportion of women who opted for increased protective measures against crime was higher than expected, while female victims with minors, the adoption of protective measures was lower than expected (Table 4).

**Table 4:** Victimization by gender and protective measuresa.

Victim of crime			Protective measures against crime		Total

			Low	High	
Women^b^	Yes	n	68	180	248
		% of group	27.4	72.0	100
		Corrected residuals	-7.6	7.6	-
	No	n	811	704	1,515
		% of group	53.5	46.5	100
		Corrected residuals	7.6	-7.6	-
	Total	n	879	884	1,763
		% of group	49.9	50.1	100
Men^c^	Yes	n	50	180	230
		% of group	21.7	78.3	100
		Corrected residuals	-6.3	6.3	-
	No	n	665	861	1,526
		% of group	43.6	56.4	100
		Corrected residuals	6.3	-6.3	-
	Total	n	715	1,041	1,756
		% of group	45.3	54.7	100

^a^ χ^2^ = 94.828, p < 0.001; φ = -0.164, p < 0.001; V = 0.164, p < 0.001

^b^ χ^2^ = 58.124, p < 0.001; φ = -0.182, p < 0.001; V = 0.182, p < 0.001

^c^ χ^2^ = 39.492; p < 0.001; φ = -0.150, p < 0.001; V = 0.150, p < 0.001

## DISCUSSION

People victimized in the last twelve months evaluate their municipality as unsafe or very unsafe. These results are consistent with those obtained in previous research^10^ ^,^ ^18^ ^,^ ^21^, in the sense that the experience of victimization implies a higher perception of insecurity. However, these results, which at first seem obvious, show that more than 40.0% of non-victimized persons match the perception of insecurity in their community, in the same terms as those of victimized persons. It is inferred from these results that the victimization does not seem to be the only factor associated with the perception of insecurity. The climate of insecurity is deeply rooted in the citizens to the extent that 42.0% consider the social climate as unsafe or very unsafe. Therefore, the trigger for the perception of insecurity is not only the fact of having been a victim of a crime; the social climate of vulnerability that, in the case of having been a victim, also counts and becomes most evident. These results are convergent with those obtained by Naplava^14^, which indicate a direct relationship between crime rates and fear of victimization, in such a way that those individuals who were aware of the level of crime in the community perceived a higher risk of being victimized. In that same study, we observed an association between state indicators of crime and fear of victimization in the communities, which supports the idea that fear of becoming a victim not only has its origin in the experience with crime, but also in the fear that arouses the feeling of vulnerability. This idea, although it has not been analyzed in this study, has deep relation with the results obtained by Hanslmaier^11^, which showed that the impact of crime rates on citizens is closely related to the information that is transmitted by social interaction, media and new technologies. The victims are not only the subject of crime, but are also transmitters of that information as part of the need to express the negative experiences in their community, by communication flows established in these communities.

In relation to the perception of insecurity and victimization based on gender, the results of this research indicate that men feel greater insecurity than women, regardless if they have been victims or not (44.0% and 40.0%, respectively). In addition, the percentage of victimized men who feel greater insecurity (58.0%) is significantly higher than that of victimized women (53.0%), results that we consider interesting and deserve further exploration in future studies. We could refer to two approaches in the interpretation of these results. The first refers to gender differences, both in the assignment and victimization. From the results of ENVIPE^b^, we came to the conclusion that the most common crimes from the perspective of victims (use and sale of drugs, theft and assault) are committed mainly by men. Likewise, according to the report made by the Government of the State of Morelos^d^, in recent years, most frequent crimes are fundamentally violent and predominantly affect men who are both victims and perpetrators. In a study conducted by San-Juan et al.^17^, it was found that women were victims more often than men. However, our study shows that the percentage of victimized men and women is very similar (12.9% and 13.2%, respectively). We believe that these differences are due to two possible causes: the most visible types of crimes in different cultural contexts; and the way of measuring victimization, since many studies usually unify direct and indirect victimization, while this study only used direct victimization. Most reports, prepared in Mexico, referred to offences related to sexual abuse or violence against women both in public and private areas^4^ ^,^ ^6^ ^,^ ^b^, which brings us to a serious problem of visibility of violence against women in all its manifestations in Mexico.

The second approach refers to the protagonist role that men have in families and communities in Mexico, in the sense that they are primarily responsible for tasks related to the safety and protection of families^9^ and, therefore, they have greater access to information about the climate of insecurity in the community. In addition, men are more involved in regulated social exchanges regulated, while women have greater weight in informal activities, thus reinforcing the man’s hegemonic role in the protection of the family and the community against crime.

Concerning the restrictions in everyday activities, it has been observed that those who have been victims of a crime reduce their daily routines substantially and, to a greater extent, men more than women (81.0% and 59.0%, respectively). These findings are in line with previous studies, in the sense that a consequence of victimization is the reduction of activities, because victims consider them a greater risk^1^ ^,^ ^8^ ^,^ ^24^.

The most important changes in daily routines of victims are related to social interaction and activities in public areas considered at risk^5^ ^,^ ^7^ ^,^ ^8^ ^,^ ^22^. These limitations increase the social isolation of victims, which is associated with the lack of social support, which could be considered a second victimization due to the effects it has on individual and social quality of life^23^. In this sense, Carvalho and Lewis^3^ emphasize that inhibition of social behavior increases the fear of being a victim, which reinforces the behaviors that involve a loss of social interactions, thus consolidating the social isolation and the feeling of vulnerability. Recently, Vilalta^23^ found changes in the way of life of Mexican population due to crime, such as stopping going out at night for fear of being a victim of crime. These restrictions were higher in young men and women. However, we observe here that these restrictions are higher in men that in women, which is attributable to the fact that, according to data from ENVIPE^b^, men are primarily involved in crime, both in the role of victims and perpetrators. One possible explanation for this discrepancy may be attributable to everyday activities selected in the survey, insofar as they refer to crimes such as theft or assault that, as previously noted, are more common in men.

The results of this study show the relationship between victimization and protective measures based on gender. Victimized persons make high use of protective measures (75.0%), compared to non-victimized ones (51.0%). It confirms the results obtained by other researchers^16^, also in high crime contexts^18^. We emphasize that the percentage of non-victimized persons adopting protective measures is high, indicating that the use of protective measures is closely related to the perception of insecurity and fear of victimization. Similarly to what happens with restrictions of everyday activities, men, especially those who were victims of crime, adopt more protective measures than women. As noted by San-Juan et al.^17^, the family protection is, still, a responsibility assigned mainly to men. In this sense, being a victim of crime can involve a failure in the task of protecting yourself and your family^20^.

Recent studies in Mexico indicate that despite the implementation of recent public policies, such as the rescue of public spaces or plan to combat drug trafficking, the failure of these actions in reducing levels of victimization and perception of insecurity requires rethinking the direction of new public policies^12^ ^,^ ^23^. Also, they have proposed measures such as: greater police presence in the area; active surveillance of members of the community; use of surveillance cameras; investment in street lighting; and implementation of activity and employment programs for young people^15^. In light of our data, we estimate that it is important that public policies not only have an impact on the prevention of crime, but also promote measures to achieve better quality of life, from the strengthening of trust between citizens and governmental institutions in public spaces, thus enhancing community coexistence and social cohesion^2^ ^,^ ^19^. At the same time, the implementation of effective public policies that reduce the levels of impunity, strengthening the perception that the Government, from the criminal justice system, punishes these behaviors would be of great importance^13^.

Some limitations in this study demand caution on conclusions drawn from it, even though its exploratory nature can serve as a basis for future research seeking to deepen the issues discussed here. Firstly, one of the most common difficulties when investigating on crime and victimization is that participants often avoid sharing certain information for fear of reprisals. Secondly, the correlational nature of the study prevents placing antecedents and consequences accurately. In this sense, incorporating the temporal dimension in future research would be interesting. Finally, it would be important to include the gender perspective with sensitive instruments that allow a greater and more rigorous assessment of violence against women.

## Footnotes

a Aguayo Quezada S, editor. Atlas de la seguridad y violencia en Morelos. Morelos, Mx: Universidad Autónoma del Estado de Morelos; 2014.

b Instituto Nacional de Estadística Geografía e Informática. Encuesta Nacional de Victimización y Seguridad Pública. Ciudad de México: INEGI; 2014.

c Elashoff JD. nQuery Advisor Version 6.01 user´s guide. Los Angeles: Statistical Solutions; 2005.

d Gobierno del Estado de Morelos. Agenda estadística anual del Poder Ejecutivo. 2014 [cited 2014 Aug 25]. Available from: http://www.transparenciamorelos.mx/sites/default/files/Ejecutivo_Central/Hacienda/oja5/Archivo%20completo%20Agenda%20Estad%C3%ADstica_0.pdf
